# Multiple Mechanisms to Regulate Actin Functions: “Fundamental” Versus Lineage-Specific Mechanisms and Hierarchical Relationships

**DOI:** 10.3390/biom15020279

**Published:** 2025-02-13

**Authors:** Taro Q. P. Uyeda, Yosuke Yamazaki, Saku T. Kijima, Taro Q. P. Noguchi, Kien Xuan Ngo

**Affiliations:** 1 Department of Pure and Applied Physics, Graduate School of Advanced Science and Engineering, Waseda University, Tokyo 169-8555, Shinjuku, Japan; 2 RIKEN Center for Biosystems Dynamics Research, Yokohama 230-0045, Kanagawa, Japan; y.yamazaki.inaho@gmail.com; 3 Bioproduction Research Institute, National Institute of Advanced Industrial Science and Technology, Tsukuba 305-8566, Ibaraki, Japan; kijima.sakutaro@aist.go.jp; 4 Department of Chemical Science and Engineering, National Institute of Technology, Miyakonojo College, Miyakonojo 885-0006, Miyazaki, Japan; t-noguchi@cc.miyakonojo-nct.ac.jp; 5 Nano Life Science Institute (WPI-NanoLSI), Kanazawa University, Kanazawa 920-1192, Ishikawa, Japan; ngoxuankien@staff.kanazawa-u.ac.jp

**Keywords:** cooperativity, polymorphism, actin-binding protein, aging, biochemical regulation, dissociation rate constants, mechanical strain, nucleator, thermal and stochastic fluctuations

## Abstract

Eukaryotic actin filaments play a central role in numerous cellular functions, with each function relying on the interaction of actin filaments with specific actin-binding proteins. Understanding the mechanisms that regulate these interactions is key to uncovering how actin filaments perform diverse roles at different cellular locations. Several distinct classes of actin regulatory mechanisms have been proposed and experimentally supported. However, these mechanisms vary in their nature and hierarchy. For instance, some operate under the control of others, highlighting hierarchical relationships. Additionally, while certain mechanisms are fundamental and ubiquitous across eukaryotes, others are lineage-specific. Here, we emphasize the fundamental importance and functional significance of the following actin regulatory mechanisms: the biochemical regulation of actin nucleators, the ATP hydrolysis-dependent aging of actin filaments, thermal fluctuation- and mechanical strain-dependent conformational changes of actin filaments, and cooperative conformational changes induced by actin-binding proteins.

## 1. Introduction

**Figure 1:**
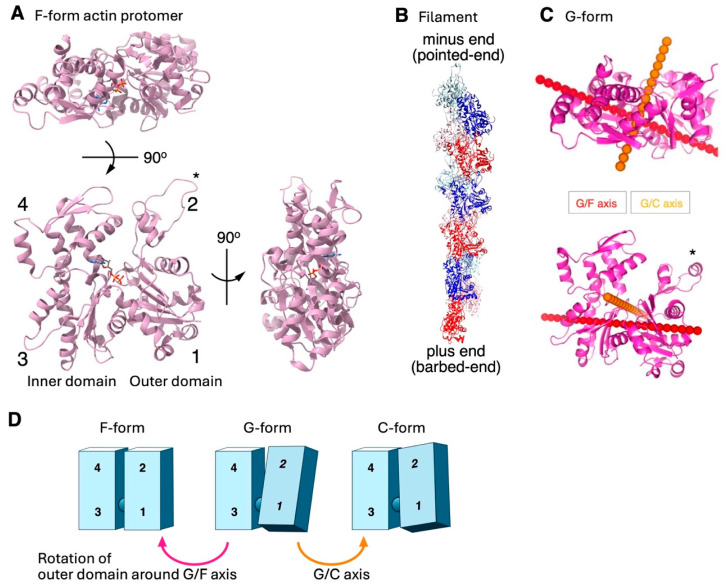
(**A**) The atomic structure of a rabbit skeletal muscle actin protomer in a filament (PDB 5JLF [7]). Actin consists of two domains, the outer domain and the inner domain. The outer domain can be divided into subdomains 1 and 2, and the inner domain into subdomains 3 and 4, as indicated. Subdomains 1 and 3 are connected by a flexible hinge involving two peptide chains, and a nucleotide (stick model), ADP in this structure, is bound at the base of the cleft between the two domains [8]. (**B**) The structure of a filament of rabbit skeletal muscle actin (PDB 3G37 [9]) with two distinct ends (the plus end or barbed-end and the minus end or pointed-end). Individual protomers are shown in different colors. (**C**) Rigid body movements of the inner and outer domains. In actin monomers, the two domains are tilted relative to one another. As actin monomers polymerize, the outer domain rotates around the G/F axis (red bar) in (**C**) that connects subdomains 1 and 3 so that the two domains are aligned parallel to one another in filaments (**A**) [10]. The two structures before and after the rotation are called the G- and F-form, respectively. As described later, actin protomers in filaments bound with cofilin have a distinct relative orientation between the two domains (C-form), involving rotation between the domains around two axes, the G/F axis and the G/C axis (orange bar). Rotation around the G/C axis allows the narrowing of the cleft [11]. The two domains move as rigid bodies during the transition between the G- and F-forms and between the F- and C-forms. These transitions are schematically shown in (**D**), except that a minor rotation around the G/F axis during the transition from G- to C-form is ignored here. Note, however, that the DNaseI binding loop or D-loop in subdomain 2, residues 200–206, and the C-terminus are flexible and adopt several different conformations, and do not necessarily participate in the rigid body movements ([12,13,14] and references therein). Thus, the D-loop in the structure shown in (**A**) is actually a loop (asterisk), but it forms a helix (asterisk) in the structure shown in (**C**), which is an actin monomer (PDB 1J6Z [15]). (**C**) is reproduced from [11], except that the arrangements are modified and an asterisk is added.

Actin is a 42 kDa square-shaped ATP/ADP-binding protein, which polymerizes reversibly to form double-helical polar filaments (Figure 1). Actin is one of the most conserved eukaryotic proteins and plays critical roles in numerous cellular functions, including cellular morphogenesis, cell motility, cell division, intracellular transport and membrane deformation in the cytosol [1]. Recent studies have revealed that actin is also involved in various nuclear functions, such as transcriptional regulation, chromatin remodeling, and DNA repair [2]. These actin functions are so essential for eukaryotic cells that it is possible to claim that these functions collectively define a eukaryotic cell. Interestingly, many of these different actin-dependent functions operate simultaneously in the cytoplasm or nucleoplasm, indicating a single species of protein that performs multiple functions simultaneously in one common milieu. Understanding the molecular mechanism of the functional differentiation of actin filaments in cells is a challenging and fundamental biological and biophysical question [3,4,5,6]. This review introduces known mechanisms that regulate actin functions in the cytoplasm and sorts them into fundamental and lineage-specific types.

## 2. Mechanisms to Create Functionally Distinct Actin Filaments

**Figure 2:**
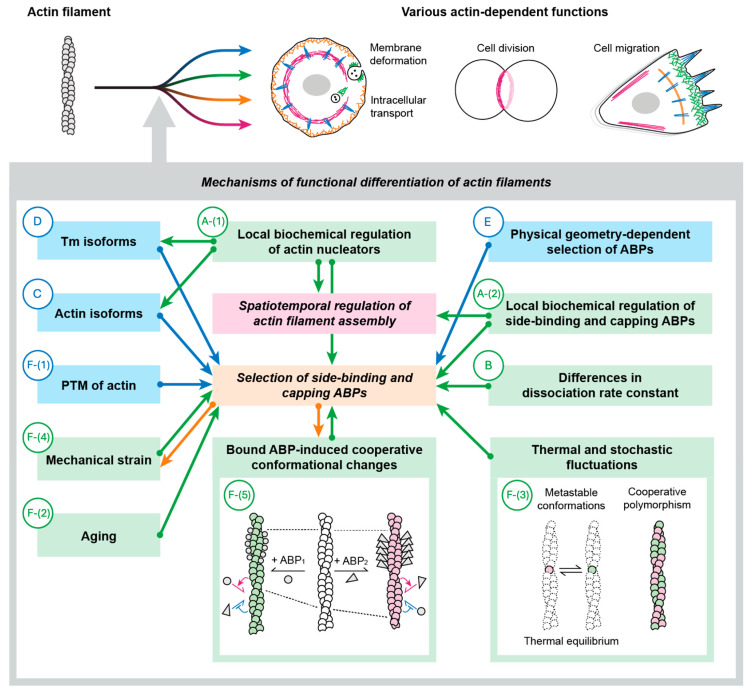
Plausible mechanisms of spatiotemporal regulation of actin filament assembly and selection of ABPs. Green shows fundamental mechanisms, whereas blue shows lineage-specific mechanisms, with arrows showing the regulatory directions. Except for thermal and stochastic fluctuations, the regulatory processes receive inputs from upstream regulators, which are not shown here.

Since each actin function depends on interactions with a specific set of actin-binding proteins (ABPs), proper cellular actin function requires the spatiotemporal regulation of actin filament assembly and the precise selection of ABPs. Plausible hypotheses to achieve these two objectives can be grouped into the following six major categories (two of which have several sub-categories) (Figure 2).

A: Local biochemical regulation of specific ABPs.
  A-(1): Local biochemical regulation of actin nucleators.
  A-(2): Local biochemical regulation of filament-side binding and capping ABPs.
B: Differences in dissociation rate constants from actin among ABPs.
C: Actin isoform-dependent selection of ABPs.
D: Tropomyosin (Tm) isoform-dependent selection of ABPs.
E: Physical geometry-dependent selection of ABPs.
F: Filament conformation-dependent selection of ABPs.
  F-(1): Post-translational modification (PTM) of actin.
  F-(2): Aging.
  F-(3): Thermal and stochastic fluctuations.
  F-(4): Mechanical strain.
  F-(5): Bound ABP-induced cooperative conformational changes.

These mechanisms are not mutually exclusive, and obviously, more than a single mechanism is needed to explain the entire repertoire of functional differentiations of actin filaments. However, this does not necessarily imply that all mechanisms are equally essential and fundamental. Presumably, some emerged later during eukaryotic evolution, particularly in the lineage of multicellular plants and animals, to support specialized actin functions required in complex multicellular organisms.

Recent metagenomic studies revealed that extant Asgard archaea and Eukarya share a common ancestor [16,17], and extant Asgard archaea contain actin that is very similar to eukaryotic actin [16,18] and a small number of primitive forms of eukaryotic ABPs [18,19]. All eukaryotes are monophyletic and descendants of a hypothetical common ancestor, referred to as the Last Eukaryotic Common Ancestor (LECA). Moreover, certain ABPs are shared by phylogenetically distant eukaryotes. Therefore, it is reasonable to assume that LECA already possessed eukaryotic actin, the conserved ABPs, and functionally differentiated cytoplasmic actin filaments. Those mechanisms are likely retained in many subsequent evolutionary lineages, so we consider those mechanisms fundamental. In this review, we evaluate the abovementioned mechanisms from this point of view.

## 3. A: Local Biochemical Regulation of Specific ABPs

The activities of many ABPs are regulated biochemically. This includes their PTM, such as reversible phosphorylation involving specific kinases and phosphatases. Reversible binding of various classes of regulators, such as small-molecule ligands and regulatory proteins, is also widespread. When those ABP regulators are localized within a cell, the activity of the regulated ABP is localized accordingly within the cell, leading to the assembly/disassembly of actin filaments at specific sites and functional differentiation of specific actin filaments within the cell.

Studies on biochemical regulation of ABP have a very long history, dating back to the discovery of Ca^2+^-dependent stimulation of the actin-myosin interaction involving the troponin-Tm complex in skeletal muscle in the 1960s by Ebashi and colleagues [20]. For localized biochemical regulation of ABPs, however, membrane-bound ligands appear more suitable than rapidly diffusible small ligands, such as Ca^2+^. Phosphatidyl inositol bisphosphate (PIP_2_) and phosphatidyl inositol trisphosphate (PIP_3_) are particularly relevant since those two lipids form complementary gradients within the cell membrane at actin-rich regions [21] and along the front-rear axis of polarized amoeboid cells [22,23,24]. If ABPs that drive lamellipodia formation are activated by PIP_3_ and those required for actin-myosin II interaction are activated by PIP_2_, this would assemble two spatially differentiated and distinctive motile units, expansive machinery in the PIP_3_-rich region, and contractile machinery in the PIP_2_-rich region, to realize unidirectional movement of the cell. Biochemical signaling pathways, both upstream and downstream of PIP_3_/PIP_2_, that regulate the actin-dependent machinery are increasingly being uncovered [25,26]. Notably, these pathways include small GTPases. Many small GTPases are anchored to the membrane and are also suitable for transmitting localized signaling. The in vitro and in vivo evidence for the biochemical regulation of ABPs is surmounting.

Below, the functional significance of the biochemical regulation of ABPs is discussed separately for ABPs required for de novo actin polymerization (nucleators) and filament-side binding/capping ABPs.

### 3.1. A-(1): Local Biochemical Regulation of Actin Nucleators

Obviously, it is critically important for cells to regulate when and where actin filaments are polymerized. In cells, spontaneous polymerization of actin is inhibited by monomer-sequestering proteins including profilin, β-thymosin and cofilin, and specific ABPs collectively called nucleators, including the Arp2/3 complex and formin family proteins, are required to assemble actin filaments from actin-profilin complexes [27]. The Arp2/3 complex initiates polymerization of new filaments from the sides of existing actin filaments to form a branched actin meshwork. Formins comprise a large family with different domain configurations, and most of them assemble unbranched actin filaments de novo while remaining bound at the growing plus end of the filament. Those nucleators require a spatial clue regarding when and where to assemble actin filaments, and this is provided by regulatory proteins, which are typically localized within a small domain inside the cell membrane. Activity of the Arp2/3 complex requires binding to regulatory proteins collectively known as nucleation-promoting factors (NPFs), which are often membrane-associated proteins [28]. WASP and SCAR/WAVE, representative NPFs, guide the localized polymerization of actin through activating Arp2/3 complexes and play critical roles in processes like cell migration [29]. Activation mechanisms of all diverse formins are not elucidated, but some are known to be activated by membrane-bound small GTPases [30].

Profilin is present in Asgard archaea [31] and conserved across most of the eukaryotic lineages [32,33]. Formins [33,34] and the Arp2/3 complex [33] are also present in all eukaryotic lineages. There are some notable exceptions, however. For example, Apicomplexan parasites lack the Arp2/3 complex [35]. This absence might seem inconsistent with the hypothesis that the Arp2/3 complex was present in LECA and inherited by all eukaryotic lineages. Instead, it could suggest that the Arp2/3 complex evolved in a specific lineage. However, this interpretation is unlikely because the absence of the Arp2/3 complex is sporadic and primarily limited to obligate parasites with specialized motility systems. This pattern, also seen with profilin and formins, strongly indicates reductive evolution rather than independent evolutionary origins. Therefore, the above general scheme, in which biochemical regulation of actin nucleators determines when and where actin filaments are assembled, appears to be fundamental. Regarding the distribution of key actin regulatory proteins in eukaryotes and their sporadic loss during evolution, we refer readers to an excellent recent review by Velle et al. [36]. Other actin nucleators such as Spire [37], Cordon-bleu [38], leiomodin [39,40], and CHUP1 [41] also contribute to the assembly of specific actin structures, but they are lineage-specific and are not fundamental. The actin nucleators are regulated by upstream factors such as NPFs for the Arp2/3 complex, but they occupy the central position at the top in the hierarchy of the actin regulatory mechanisms shown in Figure 2.

The nucleation factors do more than specify when and where to assemble actin filaments. Those issues will be discussed in the “Mechanical strain” and “Bound ABP-induced cooperative conformational changes” sections.

### 3.2. A-(2): Local Biochemical Regulation of Filament Side-Binding and Capping ABPs

Once actin filaments are assembled in the cell, the sides of the filaments are available to bind various types of side-binding ABPs. In the case of the Arp2/3 complex-nucleated filament, the plus end of the filament is available to bind plus end-binding ABP, called capping proteins.

The repertoire of side-binding ABPs is extensive, encompassing myosin motors, crosslinkers, and severing proteins. Many of those side-binding ABPs are subject to biochemical regulation. Below is a summary of what is known about the biochemical regulation of cofilin and myosin II, two representative and physiologically important side-binding ABPs, in the social amoeba *Dictyostelium discoideum*. Tm is another major side-binding ABP, but Tm is distinct in that it has been hypothesized that Tm assumes a role as the primary determinant of the function of actin structures [42]. Therefore, Tm is discussed separately in the following section.

**Figure 3:**
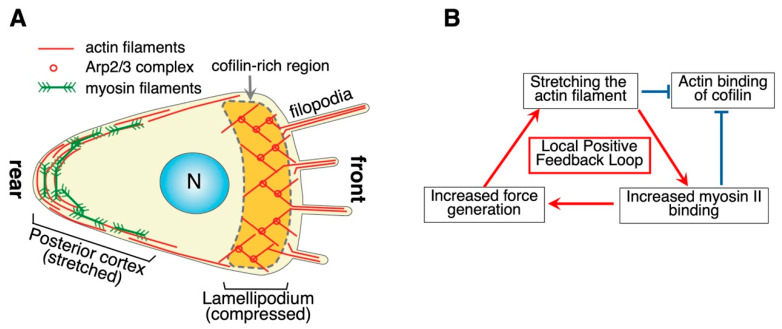
Actin structures and the regulation in a migrating *Dictyostelium* amoeba. (**A**) Schematic drawing. In the anterior lamellipodia, the polymerization force of branched actin filaments formed by the Arp2/3 complexes pushes the anterior cell membrane forward. Those actin filaments are disassembled by cofilin for dynamic turnover. In the posterior cortex, a meshwork of actin filaments interacts with filaments of myosin II to generate contractile force. Those posterior actin filaments are, therefore, mechanically stretched, while those in lamellipodia are compressed. Reproduced from [53]. (**B**) The motor domain of myosin II preferentially binds to stretched actin filaments [3]. Thus, mechanical stretching of actin filaments, increased binding of myosin II to the actin filaments, and increased tension generation would form a positive feedback loop in the posterior cortex, stabilizing the contractile property of the posterior cortex. This positive feedback loop can be activated by biochemical activation of myosin II activity, a mechanical stimulus, and stochastic fluctuation of the actin conformation. Actin binding of cofilin in the posterior region is inhibited because stretched actin filaments are resistant to cofilin binding [54] and because transient actin binding of myosin II motor in the presence of ATP inhibits actin binding of cofilin [53].

Amoeboid cells of *D. discoideum* crawl on an open substrate mainly using two distinct mechanisms in a coordinated manner (Figure 3A). The leading edge advances by pushing force generated by polymerizing actin filaments in the lamellipodia, which involves activities of Arp2/3 complexes to promote polymerization [43] and cofilin for dynamic turnover [44,45]. In contrast, the rear cortex contains actin filaments and bipolar filaments of myosin II to drive contraction [46,47], which generates hydrostatic pressure and pushes the cytoplasm forward. Filament formation of *Dictyostelium* myosin II is negatively regulated by heavy chain phosphorylation [48]. One of the specific kinases is localized along the anterior lamellipodia [49], leading to the hypothesis that this kinase plays a significant role in restricting myosin II filaments’ localization to the rear by disassembling myosin II filaments in the anterior. However, mutants lacking this kinase [49], as well as those additionally lacking both of the other two major myosin heavy chain kinases with different intracellular localizations [50], show only partial defects in intracellular localization of myosin II filaments and cell motility. Moreover, although phosphomimetic mutant myosin II cannot form filaments in vitro and in vivo and, hence, is non-functional in vivo [51], a non-phosphorylatable mutant myosin II localizes at the cell rear more prominently than the wild type, presumably due to defective filament disassembly, and is thus partially functional [51]. Therefore, turning ON and OFF myosin II temporally and spatially within a cell is not critically important for the localization and function of myosin II in *Dictyostelium*. A similar argument can be applied to the anterior localization of cofilin. The activity of cofilin is known to be regulated by three biochemical factors, i.e., phosphorylation of Ser3, pH, and binding to PIP_2_ [52]. Removing these three biochemical regulators of cofilin individually did not significantly impact cell motility (discussed in [53]), indicating that none is essential for cofilin functions.

In summary, *Dictyostelium* possesses biochemical switches capable of locally turning ON and OFF the activities of myosin II and cofilin. When the myosin II switch is fixed in the OFF state across the cell, the cell will behave as if myosin II is missing (the same type of experiment has not been reported for cofilin, presumably because cofilin is essential). However, if the switch is fixed in the ON state, the cell will behave more or less normally. To the best of our knowledge, no report has demonstrated that a constitutively active ABP is totally non-functional in vivo. The widely conserved nature of that biochemical regulation in extant eukaryotes suggests that biochemical regulation of ABPs improves the robustness and/or efficiency of the process. This may be analogous to installing switches that turn ON and OFF individual room lights to prevent wasting electricity, although the room lights are useful even if the switches are fixed in the ON state. This implies that some other mechanisms play critical or significant roles in specifying which ABPs bind to the sides and ends of specific actin filaments, thereby defining the function of the filament. We speculate that some biochemical regulations of side-binding and capping ABPs were already present in LECA, while others evolved later in specific lineages. Similarly to actin nucleators, the biochemical regulation of the side-binding and capping ABPs is at the top in the hierarchy of the actin regulatory mechanisms shown in Figure 2.

## 4. B: Differences in Dissociation Rate Constants Among ABPs

Different ABPs dissociate from actin filaments with varying dissociation rates. Imagine an extreme scenario in which there are two distinct populations of actin filaments in the cytoplasm: one that remains completely stable with no turnover, and another that cycles and turns over at a specific rate. For an ABP that dissociates from actin filaments at a rate slower than the turnover rate of the cycling filaments, the ABP molecules bound to the cycling filaments would be released at the turnover rate of those filaments. In contrast, ABP molecules bound to the stable filaments would dissociate from those actin filaments more slowly than from the cycling filaments, at their intrinsic dissociation rate. Over time, the ABP molecules would be enriched on the stable filaments. Conversely, ABPs that dissociate from actin filaments faster than the turnover rate of the cycling filaments would dissociate from both cycling and stable filaments at their own dissociation rate. It is therefore predicted that the slow dissociaters tend to accumulate in slowly cycling or relatively static actin structures. In contrast, rapid dissociaters would bind uniformly to all actin filaments in the cell [55]. Moreover, in a polarized cell with a retrograde cortical actin flow [56,57], the stable binders may ride this flow and accumulate in the posterior cortex [57,58,59,60]. Ivanova et al. (2024) synthesized a library of “designed ankyrin-repeat proteins” (DARPins) with varying actin-binding properties and expressed them in cultured mammalian cells. Interestingly, individual actin-binding DARPins exhibited different localization patterns, which were correlated with their dissociation rates from actin filaments [61].

These results indicate that the difference in dissociation rate from actin filaments can generate differential localization patterns of ABPs between slowly and rapidly turning over actin structures. However, different ABPs bind differentially to various actin structures in fixed cells and tissues [62]. Because actin filaments in fixed samples do not undergo turnover, this differential ABP binding must result from mechanisms other than the differences in dissociation rate constants.

This mechanism relies on different properties of actin filaments, i.e., different depolymerization or turnover rates. Consequently, it cannot generate distinct actin structures from homogeneous actin filaments and is therefore positioned lower in the hierarchy of actin regulatory mechanisms. The diversity of DARPins [61] suggests that random mutations can readily generate ABP variants with different dissociation rates, supporting the hypothesis that this mechanism was functional in LECA. However, this remains speculative.

## 5. C: Actin Isoform-Dependent Selection of ABPs

Although the amino acid sequences of eukaryotic actins are highly conserved, many organisms express multiple actin isoforms with slightly different amino acid sequences in one cell. Suppose different isoforms have differential affinities for different ABPs and form segregated actin structures in a cell. In that case, the actin isoforms may contribute to the functional differentiation of the actin filaments in cells. However, fundamental roles of actin isoforms to bias ABP binding can be eliminated since many unicellular organisms, including budding yeast [63], fission yeast [64], *Chlamydomonas* [65], and *Tetrahymena* [66] express only one actin species. The *D. discoideum* genome contains as many as 33 actin genes. However, 17 of them encode the same amino acid sequence, and their products comprise 95% of the total actin proteins [67]. Thus, the motility of this organism, which is often considered a model system to study amoeboid motility and cytokinesis of animal cells, depends on virtually one actin species.

Vertebrates express three distinct actin isoforms: muscle-specific α isoforms and β and γ non-muscle isoforms. The non-muscle isoforms are expressed ubiquitously. Recent analyses indicated certain differences in intracellular localization of the two non-muscle isoforms in non-muscle cells (reviewed in [68,69]), suggesting functional differences between the two isoforms. However, mice lacking γ actin grew to adulthood, albeit with several problems [70]. Homozygous disruption of β actin loci was embryonic lethal in mice, but development proceeded normally to embryonic day 8.5 [71]. Moreover, mice expressing γ actin from the mutated β actin locus, as well as cells derived from them, were normal and displayed no sign of deficiency in the absence of the β actin protein [72]. These results are consistent with the idea that the distinction between the two non-muscle isoforms is not critically important at the single-cell and organism levels. Vedula et al. [72] provided data suggesting that the different nucleotide sequences of β and γ actin mRNAs, rather than the different amino acid sequences, are important for normal physiological functions.

*Arabidopsis thaliana*, a model organism of seed plants, expresses eight actin isoforms, of which ACT2, ACT7 and ACT8 are the vegetative isoforms expressed ubiquitously in vegetative tissues [73]. Purified ACT2 and ACT7 exhibited distinct biochemical properties [74], and defective root hair growth in ACT2-KO plants was not complemented by overexpression of ACT7 [75], indicating that ACT2 and ACT7 are not functionally interchangeable. Nonetheless, overexpression of a single ACT8 isoform was sufficient for normal plant development [75], indicating that isoform-dependent functional differentiation of actin filaments is not essential in the vegetative tissues of *A. thaliana*.

The distinction among actin isoforms presumably emerged to support highly divergent actin functions required in complex multicellular organisms. Muscle-specific isoforms (α-actins) in vertebrates and possibly the pollen-specific reproductive actins in *A. thaliana* may represent exceptions, as they appear to have evolved for essential roles in highly specialized actin functions. Alternatively, these specific actin isoforms might have co-evolved with distinct sets of ABPs tailored to specialized tissues. In *A. thaliana*, overexpression of the reproductive actin ACT1 in vegetative tissues was highly toxic. However, co-expression of reproductive profilin or ADF effectively suppressed the toxic phenotypes [76]. This observation suggests that the functional distinction between vegetative and reproductive actin isoforms may not be as pronounced as previously thought. It remains to be determined whether ACT1, when co-expressed with its corresponding regulatory ABPs, can fully compensate for the loss of vegetative actin isoforms in vegetative tissues.

Another important question regarding actin isoforms is how different actin isoforms assemble distinct actin structures in a cell. Studies have shown that different isoforms of vertebrate formin polymerize filaments of a specific actin isoform (β and γ actins) both in vitro [77] and in vivo in HeLa cells [69,77]. In myoblasts and myofibrils, high levels of α actin are expressed in addition to β and γ actins. The α isoform is specifically incorporated into the thin filaments of sarcomeres, whereas the β/γ isoforms are integrated into cytoplasmic actin structures [78]. This presents another case of striking segregation of actin isoforms within a cell. Although the mechanisms underlying this segregation are not fully elucidated, muscle-specific actin nucleators such as a formin FHOD3 and isoforms of leiomodin have been identified [39,40], and they may play roles in the assembly of α actin-containing thin filaments. Moreover, fluorescently tagged ACT2 and ACT7 formed distinct structures in seed plant cells [79], suggesting that plant cells also utilize actin isoform-specific actin nucleators to assemble actin isoform-specific actin structures. These findings indicate that the actin isoform-dependent selection of ABPs mechanism is under the regulation of actin nucleators.

## 6. D: Tropomyosin (Tm) Isoform-Dependent Selection of ABPs

Competition for binding sites on an actin protomer restricts the binding of multiple ABP molecules to one actin protomer. However, when competition for binding sites among different ABPs occurs independently on individual actin protomers, this would not result in preferential binding of one or a small number of ABP species to a stretch of the filament, which is required for functional differentiation of the actin filament. One scenario in which the binding of one ABP to an actin protomer could lead to functional differentiation of a filament or a segment of a filament is when the binding of the first ABP to one actin protomer revokes cooperative conformational changes along the actin filament, mediated by mechanisms that will be discussed later. Another scenario involves a single ABP or a chain of interconnected ABP molecules simultaneously regulating the ABP binding specificity of multiple actin protomers, as observed in the case of Tm.

Tm is a long, dimeric coiled coil, and when bound to an actin filament, one Tm dimer spans several (typically seven) actin protomers along one protofilament. Thus, Tm is a unique ABP in that one Tm dimer can simultaneously regulate the ABP binding of several consecutive actin protomers. Tm was initially identified as a component of striated muscle [80]. In striated muscles, Ca^2+^ triggers azimuthal movement of a Tm dimer in a troponin-mediated manner, exposing the myosin binding sites on seven consecutive actin protomers and stimulating the actin-myosin interaction [20,81]. Later, Lazarides discovered that mammalian non-muscle cells also contain Tm [82], and it is now known that vertebrates contain over 40 different variants of Tm protein from 4 genes through alternative splicing and PTM (e.g., [83]). In this article, we collectively refer to different Tm proteins that are encoded by different Tm genes or generated by alternative splicing and PTM “isoforms”. By analogy to muscle Tm, a non-muscle Tm dimer may simultaneously regulate the ABP binding preference of several consecutive actin protomers. If binding of Tm to an actin filament is cooperative, as has been demonstrated for muscle Tm [84,85], and this cooperativity can discriminate among different isoforms of non-muscle Tm that are co-expressed in one cell, the entire length or segments of actin filaments are expected to be associated with a specific isoform of Tm. This isoform-specific differential actin binding and cluster formation of Tm was demonstrated in vitro [86]. Clustering of a Tm isoform along a section of an actin filament and the resultant selective binding of other specific ABPs may result in functional differentiation of a stretch of an actin filament.

Indeed, in multicellular animal cells, multiple non-muscle Tm isoforms segregate from one another in one cell, often accompanying the localization of a particular ABP (reviewed in [42]). Overexpression of the Tm3.1 isoform in a rat neuronal cell line recruited myosin II to stress fibers while inhibiting the binding of ADF/cofilin to the actin filaments. In contrast, overexpression of the Tm1.12 isoform promoted ADF/cofilin binding to actin filaments to generate filopodia that do not contain myosin II [87,88]. Moreover, certain Tm isoforms can distinguish and recruit a specific isoform of myosin (e.g., myosin I, II and V, reviewed in [89]). These studies led to the hypothesis that Tm is the “master regulator” of ABP binding and, hence, the function of actin filaments in non-muscle cells [83].

However, it is questionable whether Tm should be considered a fundamental regulator of actin filament functions. Fungi, including budding and fission yeasts, also have Tm (one Tm gene in fission yeast and two in budding yeast). In contrast, plants and many single-cell eukaryotes, including *D. discoideum, Entamoeba histolytica*, *Trypanosoma brucei*, and *Tetrahymena pyriformis*, do not have Tm (for the distribution of Tm in Eukarya, see an excellent review from an evolutionary point of view by Gunning et al. [90]). This makes it highly unlikely that LECA possessed a Tm. Rather, Tm presumably emerged in the common ancestor of fungi and Metazoa, and formed a large family during the evolution of complicated multicellular animals. This precludes the position of Tm as a fundamental regulator of the functions of actin.

*Dictyostelium* amoeba performs several actin-dependent motile activities including amoeboid motility driven by lamellipodia-dependent anterior expansion and myosin-dependent posterior cortical contraction, phagocytosis, contractile-ring-dependent cytokinesis, and even multicellular development [91]. Moreover, *Dictyostelium* amoeba was recently shown to switch to a bleb-dependent motility mode when mechanically constrained [26], similar to malignant cancer cells [92,93]. These activities are dependent on interactions of actin with ABPs that are shared with vertebrates, including myosin motors of classes I, II and V, actin nucleators such as the Arp2/3 complexes, ten formins and SCAR, crosslinkers such as α-actinin, fimbrin and filamin, cofilin and its regulators such as coronin and Aip1, adhesion-related talin and paxillin, and profilin, but notably, without Tm. This indicates that many of the actin-dependent motile activities characteristic of animal cells with many Tm isoforms can be accomplished without Tm.

What, then, are the roles Tm plays in yeasts and vertebrates? Knock-out experiments demonstrated that Tm or a specific isoform of Tm is necessary for a particular actin function in some instances. For example, disruption of the single Tm gene (*cdc8*) in fission yeast was lethal because cells could not assemble contractile rings [94]. This phenotype is consistent with the idea that Tm specifies the function of actin filaments in contractile rings. However, the defect of contractile ring assembly in a temperature-sensitive *cdc8* mutant was partially suppressed at the restricted temperature by a mutation in Adf1, a homolog of cofilin [95]. Budding yeast has two Tm isoforms [96,97], which were recently shown to be functionally redundant [98]. Deleting *TPM1*, the major isoform of Tm, caused a mild growth defect accompanying the loss of actin cables [96]. Again, however, that defect was suppressed by simultaneous deletion of Aip1, a collaborator of Adf/cofilin [99]. Considering the actin-protective effect of Tm from severing and depolymerization by ADF/cofilin [100,101,102,103,104], including in fission yeast [95], these genetic studies employing both fission and budding yeasts suggest that the critical role of yeast Tm is to regulate the disruptive activities of Adf/cofilin and ensure the proper stability and dynamics of actin filaments. In yeasts, at least, the role of Tm in determining the function of a specific group of actin filaments does not appear to be essential. This idea is further supported by the work of Johnson et al. [105], which will be discussed below. In other words, the actin regulatory system in cells that evolved with Tm depends on Tm, but proper functional differentiation of actin filaments can be realized without Tm if the abundance and specific activities of other ABPs are appropriately tuned. This is probably why *Dictyostelium* amoeba can perform various actin-dependent functions without Tm.

Homozygous deletions of each of three mouse Tm genes were lethal, and the lethality was not complemented by the expression of other Tm genes [106]. Thus, each isoform of mouse TMs performs non-redundant, essential functions. Moreover, cell biological studies demonstrated that mammalian Tm isoforms play a significant role in specifying which ABP to bind to specific sets of actin filaments [83]. However, considering the lessons from the yeast genetics studies, more in-depth evidence is needed for a mechanistic understanding of how Tm isoforms specify ABP binding in vertebrates.

Another important question regarding Tm is how a specific Tm isoform is recruited to particular sets of actin filaments. Again, yeast molecular genetic studies provide an important clue. Fission yeast cells have three distinct actin structures, actin patches, actin cables, and contractile rings, each assembled by distinct actin nucleators (two different formins and the Arp2/3 complex). Actin filaments in actin patches have no bound Tm, those in longitudinal actin cables assembled by formin For3 bound with unmodified Tm, and those in contractile rings assembled by another formin Cdc12 bound with acetylated Tm. Elegant molecular genetic studies using mislocalizing chimeric formins demonstrated that the formin isoform determines which Tm isoform associates with the actin filament it assembles [105]. Consequently, Gunning et al. proposed possible mechanisms by which a specific formin isoform dimer at the filament plus end can recruit a specific isoform of Tm along the length of the filaments [83]. In this scenario, Tm is more like a middle manager working under the direction of formins, rather than a master regulator. Notably, in Johnson et al.’s study, despite the mislocalization of formins and Tm isoforms, myosin II and myosin V were properly localized along the contractile rings and actin cables, respectively, and the mutant cells were viable. Those results indicate that, at least in fission yeast, Tm isoforms cannot dictate which ABP can interact with the actin filaments they are bound along, casting doubt on the competence of Tm as the middle manager in ABP sorting.

## 7. E: Physical Geometry-Dependent Selection of ABPs

An intriguing question that arises from this mutant yeast with mislocalizing formins and Tms [105] is how myosin II and V are able to bind to the correct actin filament structures in mutant cells, despite the incorrect guidance provided by Tm. A plausible explanation lies in the differences in actin filament organization: actin cables are thin bundles of filaments with uniform polarity [107], while contractile rings are significantly thicker bundles with mixed polarities [108]. It may well be that myosin II filaments prefer to bind thick actin filament bundles with mixed polarities, whereas myosin V prefers to bind bundles of uniform polarity. In support of this model, an in vitro reconstitution study demonstrated that myosin II filaments selectively contracts and disassemble actin filaments with mixed polarities [109]. In contrast, a team of myosin V motors on one liposome was shown to move more steadily on oriented actin filaments with a uniform polarity in vitro [110]. Moreover, formation of normal actin cables in fission yeast depended on myosin V activity [111], suggesting an active role of myosin V in forming actin cables. Myosin X, which is localized at the tips of filopodia in animal cells, also appears to recognize its track based on the geometry of actin filaments. This is evident from its preference for moving along parallel bundles of actin filaments formed by either fascin or methylcellulose in vitro [112].

Likewise, physical geometry of actin filaments has been shown to affect binding of specific ABPs. In certain cases, the physical size of an actin-binding protein (ABP) or its complex can influence its ability to bind actin. For instance, the diffusion of large ABP complexes may become rate-limiting, particularly in viscous environments. Truong et al. (2021) provided an intriguing example, showing that the physical pore size of the cortical actin filament meshwork restricts the diffusion of myosin II mini-filaments into the cortex, thereby affecting contractility [113].

Another case in which the size of ABP matters, relative to the geometry of actin filaments, is the bundling of actin filaments. Certain ABPs (e.g., α-actinin, fascin, and fimbrin (plastin)) have two actin binding sites and crosslink actin filaments to form parallel bundles. Due to different distances between the two actin binding sites within one crosslinker, those bundling ABPs crosslink actin filaments at different spacings. When one bundler crosslinks two actin filaments, the same bundler can readily join, but it is predicted that other bundlers with a different interfilament spacing are excluded due to a mismatch of the interfilament distance. Winkelman et al. [114] demonstrated that fascin (interfilament spacing = ~8 nm) and α-actinin (interfilament spacing = ~35 nm) form mutually exclusive bundles when they co-exist with actin filaments both in vivo and in vitro. Fimbrin, with a short interfilament spacing, was able to join the fascin-assembled bundles but not α-actinin-assembled bundles. Fission yeast α-actinin and fimbrin segregate to different bundle structures both in vivo and in vitro [115], and part of this may be due to the above interfilament spacing-dependent segregation. The segregation of α-actinin and fimbrin in fission yeast will be discussed again in the “Bound ABP-induced cooperative conformational changes” section.

The ABPs mentioned in this section, i.e., myosin II, V and X, and a pair of bundler with different interfilament spacings, are not present in all major lineages of Eukarya [36]. Therefore, the physical geometry-dependent selection of ABPs mechanism involving ABPs discussed here is unlikely to be present in LECA and is not considered fundamental. The segregation of myosin II and V and the selective penetration of myosin II mini-filaments into the cortex depends on the geometry of the pre-assembled actin structures. Although bundling ABPs can assemble appropriate bundle structures without upstream regulators in vitro, they should be subject to regulation by other mechanisms in vivo, as discussed above. We thus consider that this category is low in the hierarchy of actin regulatory mechanisms.

## 8. F: Filament Conformation-Dependent Selection of ABPs

The most well-known case of the conformation of actin filaments affecting the binding of a specific ABP is the preference of cofilin to bind to ADP-actin filaments over ATP- or ADP·Pi-actin filaments [116]. Binding of the actin-binding domain (ABD) of dystrophin to actin filaments was also shown to depend on the conformation of the actin filaments [117].

Moreover, many ABPs have been shown to bind cooperatively to actin filaments, based on sigmoidal binding curves and/or indirectly based on the formation of clusters along actin filaments. Those cooperatively binding ABPs include Tm [85], coronin 1-C [118], dystrophin [119], α-catenin [120], myosin II motor fragments [121,122,123], fimbrin ABD2 [124], and drebrin [125]. The ABP clusters can form through two distinct mechanisms. The first involves direct binding between ABP molecules along an actin filament. For example, Tm dimers bind to each other in a head-to-tail manner, forming a polymer even in the absence of actin filaments [126,127]. This intrinsic affinity between Tm dimers enables the formation of Tm clusters along actin filaments. For cooperative actin binding and cluster formation of fission yeast Tm [104,128] and vertebrate muscle and non-muscle Tm isoforms [129], the contribution of direct contact between Tm ends was suggested [85]. Similarly, the motor domains of muscle myosin II, known as subfragment 1 or S1, physically interact along the protofilaments of fully “decorated” actin filaments [130]. The binding affinities between motor domains may drive the clustering of motor domains along actin filaments. The second mechanism relies on the propagation of ABP-induced conformational changes to neighboring actin protomers (cooperative conformational changes), which increases the affinity for that ABP, and an increasing number of ABP molecules bind to their neighbors to form clusters. Except for the myosin motor domain and Tm, ABDs of ABPs bound along an actin filament do not touch each other, and hence, should depend on the latter mechanism. The heavy meromyosin (HMM) of myosin II forms clusters along actin filaments in the presence of a low concentration of ATP. Under these conditions, HMM binding is sparse, with several unbound actin protomers typically separating the bound HMM molecules, such that HMM molecules in clusters do not physically contact one another [122]. Thus, this cooperative binding should also depend on cooperative conformational changes of actin filaments. Therefore, cluster formation of most of the cooperatively binding ABP should involve ABP-induced cooperative conformational changes of actin filaments. Those ABPs can induce cooperative conformational changes to actin filaments, and conversely, their actin-binding affinity is affected by the conformation of the actin filaments. Cluster formation by those ABPs will be discussed in more detail in the following “Bound ABP-induced cooperative conformational changes” section.

To evaluate the binding affinity between an ABP and a specific actin filament, it is more straightforward to use just the ABD of an ABP, rather than the full-length ABP. The pioneering work by Washington and Knecht [131] demonstrated that the GFP-ABD of α-actinin was localized in the anterior pseudopod, while that of filamin was localized in the posterior cortex in polarized *Dictyostelium* cells, even though both ABDs are homologous in that both consist of two tandem calponin homology domains (CHDs). The localization of these ABDs matched with that of the parent full-length ABP molecules. Subsequently, the same group presented evidence indicating that the posterior localization of GFP-filamin ABD depends on unexpected dimerization of this fusion protein, which would slow the dissociation from actin filaments, causing accumulation to the more stable posterior actin filaments (i.e., dissociation rate-dependent sorting) [55]. However, Shibata et al. [132] demonstrated that filamin ABD has a specific affinity for the posterior actin filaments, based on rapid translocation of free filamin ABD to the posterior cortex. This conclusion suggests that the structure of posterior actin filaments differs from that of the anterior pseudopod. Utrophin is another ABP that contains an ABD of tandem CHDs. Harris et al. generated a panel of point-mutant utrophin ABDs, which showed different localizations in vivo and binding preferences in vitro [133]. That study indicated that subtle differences in the amino acid sequence can profoundly affect binding preferences.

Vertebrate talin is an ABP typically found in focal adhesions, with an ABD containing the I/LWEQ motif. *Dictyostelium* has two talin homologs, and in migrating *Dictyostelium* amoeba, talin B is localized in the anterior region, while talin A is localized in the posterior cortex. Tsujioka et al. showed that just the GFP-ABD of talin B is also localized in the anterior region, while the GFP-ABD of talin A is localized in the posterior cortex [134]. Motor domains of different myosin isoforms also show distinct intracellular distributions. Differential intracellular localizations of talin ABDs and myosin motor domains are other examples of homologous ABDs showing distinct intracellular localizations, and will be revisited in the “Mechanical strain” section.

Hereafter, we explore five distinct mechanisms by which the conformation of actin filaments influences the binding preferences of various ABPs or ABDs. They are the following: (1) Post-translational modification (PTM) of actin, (2) aging, (3) thermal and stochastic fluctuations, (4) mechanical strain, and (5) bound ABP-induced cooperative conformational changes. As will be discussed in detail, aging, thermal and stochastic fluctuations, and mechanical strain-dependent conformational changes of actin protomers appear to be inherent properties of actin, as they are associated with its ATPase activity or the intrinsic flexibility of the filaments. This leads us to speculate that those mechanisms are fundamental. Regarding ABP-induced cooperative conformational changes—most notably those induced by cofilin—cofilin is ubiquitous in Eukarya, suggesting that actin filaments in LECA were likely regulated by cooperative conformational changes induced by cofilin and other ABPs it possessed. However, our knowledge on conformational changes of actin filaments is based primarily on studies of skeletal muscle α actin. More comparative studies using actins from various extant species, including Asgard archaea, are needed to validate our hypothesis that filament conformation-dependent selection of ABPs is a fundamental mechanism of actin regulation. One more point to note before going into detail is that actin filaments are not static, rigid structures; rather, filament structures fluctuate significantly, both temporally and spatially, due to thermal dynamics [135,136,137,138,139]. This is a critical point of view, different from the impressions obtained when viewing high-resolution structures revealed by cryoelectron microscopy (cryo-EM).

### 8.1. F-(1): Post-Translational Modification (PTM) of Actin

Actin undergoes a variety of PTMs, some of which were shown to affect the functions of actin (reviewed in [140]), suggesting the possibility that those PTMs bias ABD binding.

Among the many actin PTMs, the N-terminal processing of actin polypeptide is well known. Although this PTM is widespread, it is not universal among eukaryotes [141]. Moreover, this modification is irreversible and, hence, unlikely to contribute to dynamic reorganization of the actin cytoskeleton. Recently, actin depolymerization induced by MICAL-dependent reversible actin oxidation was demonstrated [142,143]. However, MICAL genes have been found only in *Drosophila* and vertebrate genomes [144] and cannot be fundamental. Reversible functional and structural regulation of actin by phosphorylation of Tyr53 was found in *Dictyostelium* actin [145,146]. Phosphorylation of Tyr53 was also found in rat neurons [147], but the universality of this PTM has not been demonstrated. Currently, no evidence indicates that PTM regulation of actin is fundamental.

### 8.2. F-(2): Aging

It is well established that in physiological actin polymerization, ATP-containing G-actin molecules are added to the plus ends of existing actin filaments. The bound ATP is then hydrolyzed, and the resultant Pi is released to yield an ADP-containing actin protomer. Because ATP hydrolysis and Pi release proceed with certain rate constants (0.3 s^−1^ [148] and 0.003 s^−1^ [149], respectively for muscle α actin), it is generally assumed that actin protomers near the plus end carry ATP, followed by a segment rich with ADP·Pi-carrying protomers, and those near the minus end only have ADP. This process, often called “aging” of actin filaments, has profound physiological consequences since cofilin preferentially binds to the ADP-bound actin protomers [116] and disassembles the aged filaments. Moreover, myosin V travels longer distances along ADP·Pi-actin filaments than along ADP-actin filaments in vitro, whereas myosin VI exhibits the opposite behavior, running longer along ADP-actin filaments than along ADP·Pi-filaments [150]. This distinction may have physiological significance, as myosin V moves toward the plus ends of actin filaments, where ADP·Pi-protomers are predominant, while myosin VI moves toward the minus ends, where ADP-protomers are more abundant.

How does cofilin distinguish ADP-actin filaments from ATP- or ADP·Pi-filaments? The presence or absence of γ phosphate or a released phosphate ion in the nucleotide-binding pocket could profoundly impact the structure of an actin protomer. However, recent cryo-EM failed to detect conformational differences between ADP- and ADP·Pi-actin filaments [151,152]. This point will be discussed in the next section.

Caldesmon is a calmodulin-binding ABP involved in the regulation of smooth muscle contraction. Biochemical analysis using a caldesmon ABD suggested that maturation of polymerizing actin filaments proceeds through two intermediate conformations, with the time course several-fold slower than ATP hydrolysis and Pi release [153]. These intermediate states may correspond to the two “tilted states” that Galkin et al. observed by cryo-EM [154] in nascent actin filaments [155,156]. This possibility may be worth examining in greater detail since the half-life of dynamic actin filaments in non-muscle cells is very short, in the range of minutes (e.g., [58,157,158]), and most of the dynamic actin filaments may be in the intermediate states that are detected by the caldesmon fragment, rather than the stable, aged ADP-containing filaments, which have been extensively characterized.

### 8.3. F-(3): Thermal and Stochastic Fluctuations

**Figure 4:**
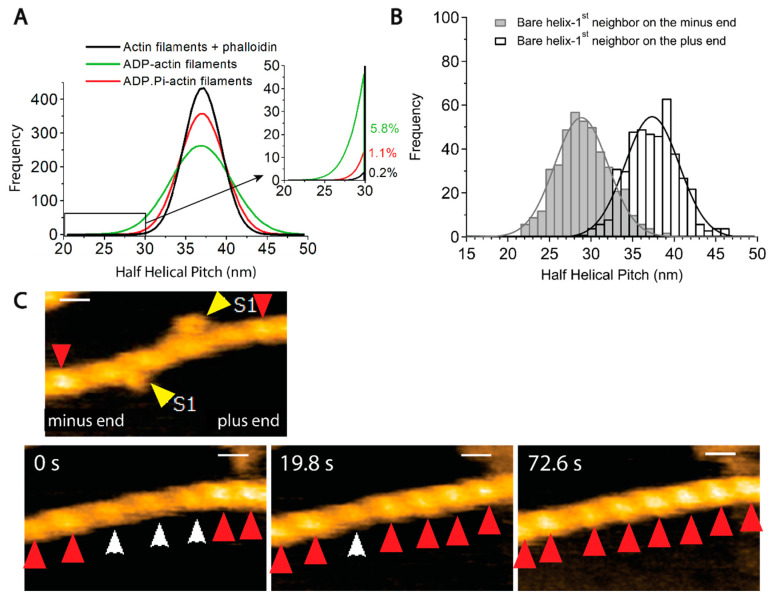
HS-AFM of actin filaments in the absence and presence of cofilin. (**A**) Histograms illustrating the distribution of the HHP of actin filaments under three conditions: phalloidin-stabilized actin filaments (37.0 ± 2.3 nm; mean ± SD), ADP-actin filaments (36.9 ± 3.8 nm), and ADP·Pi-actin filaments (37.0 ± 2.8 nm). The enlargement on the right shows fractions of the naturally supertwisted half helices with HHPs shorter than 30 nm. (**B**) Histogram of HHP of bare half helices on the minus-end and plus-end side of cofilin clusters. Half helices on the minus-end side of cofilin clusters have a supertwisted HHP (C-form like), whereas those on the plus-end side have a normal HHP (F-form). (**C**) HS-AFM image of an actin filament with two cofilin clusters. Transient binding of S1 of muscle myosin II indicated that the minus end of the filament is to the left, and the plus end to the right (top image). The lower three still images show the progressive growth of two cofilin clusters. Cofilin clusters can be identified by the brighter color of the crossover points (red arrows) due to increased thickness, reflecting bound cofilin molecules. White arrows show crossover points of bare sections. The cluster on the right grew in the direction of the minus end, whereas the cluster on the left did not grow toward the plus end. (**A**) was reproduced from [138] and (**B**,**C**) from [159].

Egelman et al. discovered that half helical pitches (HHP) of negatively stained actin filaments in electron micrographs are variable and conjectured that helical pitches fluctuate due to thermal motion [135]. This view was subsequently confirmed by real-time imaging using high-speed atomic force microscopy (HS-AFM) [159]. More recent HS-AFM analyses revealed variations in axial distance between actin protomers and the number of actin protomers in one HHP [138]. This latter study also found that the average HHP is the same between ADP-actin filaments and ADP·Pi-actin filaments, consistent with the cryo-EM studies [151,152]. Notably, however, the variance of the HHP distribution of ADP-actin filaments was much broader than that of ADP·Pi-actin filaments (Figure 4A), consistent with earlier mechanical measurements, which revealed that ADP·Pi-actin filaments are stiffer to bend than ADP-actin filaments [160].

This finding is particularly relevant to understanding why cofilin prefers to bind ADP-actin filaments. Cofilin binds cooperatively to actin filaments, forming tight clusters [159,161,162]. The helical pitch of an actin filament in cofilin clusters is shortened by 25% [161]. Galkin et al. [163] proposed that, when a cofilin cluster is formed, cofilin molecules first recognize sections of actin filaments with a naturally shortened helical pitch, bind there, and stabilize the supertwisted structure. If the supertwisted structure within the nascent cofilin cluster is propagated to neighboring bare sections of the filament, new cofilin molecules in solution will bind there, growing the cofilin cluster. HS-AFM revealed that the supertwisted structure in the cofilin cluster is actually propagated to the neighboring bare zone on the minus end side (Figure 4B,C) [159], providing experimental support for this model (note, however, that a cryo-EM study failed to detect the propagation of the supertwisted structure to the neighboring bare zone [164], and the reason for this discrepancy is unknown at this moment). Within this framework, the observation that helical pitches of ADP·Pi-actin filaments fluctuate within a narrower range indicates that the chances of cofilin molecules in solution to find naturally supertwisted segments along the ADP·Pi-actin filaments are much smaller than along ADP-actin filaments. Ngo et al. (2024) proposed that this is why cofilin preferentially binds to ADP-actin filaments.

**Figure 5:**
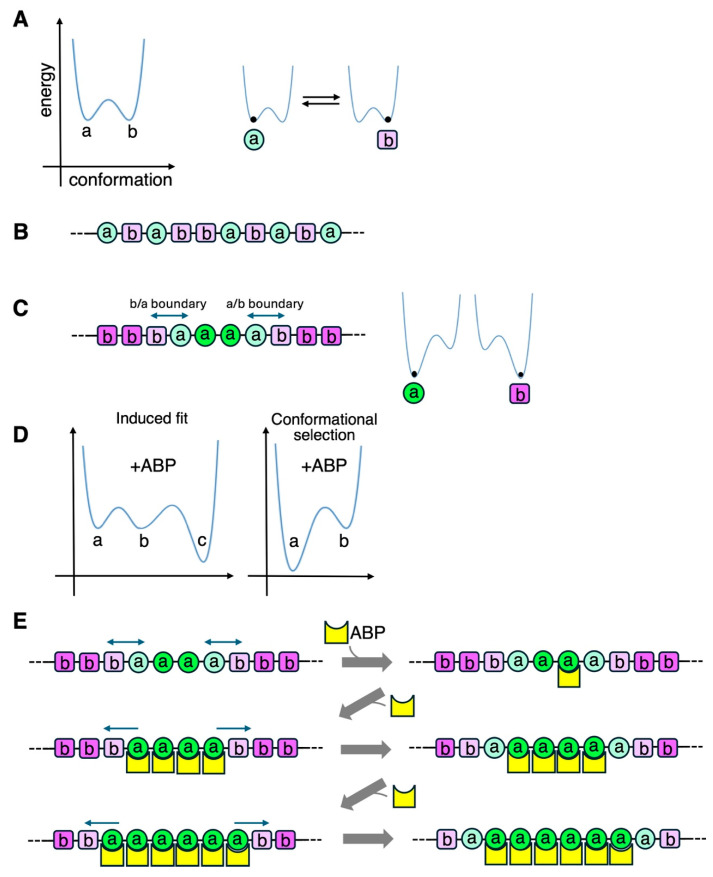
A schematic model for ABP-induced cooperative conformational changes propagated along an actin filament. (**A**) For simplicity, it is assumed that each actin protomer has two equal metastable conformational states, shown as a light green circle and a light purple square, respectively, that are in thermal equilibrium. (**B**) In a hypothetical filament in which protomers do not affect each other’s conformation, each protomer assumes either the “a” or “b” conformation and goes back and forth stochastically due to thermal fluctuation. For further simplicity, an actin filament is represented by a single protofilament. (**C**) In real actin filaments, adjoining actin protomers tend to assume the same conformation (cooperative polymorphism). As discussed below and in the main text, cooperative polymorphism of actin filaments is supported by cooperative binding and cluster formation by various ABPs. Here, however, we presume that the cooperative polymorphism is an ABP-independent inherent property of actin filaments, which is supported by a cryo-EM study [166]. The stabilization of a specific conformation is manifested by a deeper potential well, shown on the right by a dark green circle and a dark purple square, respectively. Here, it is further assumed that the stabilization of a specific conformation occurs only when both neighbors are in the same conformation. In other words, unidirectional propagation of conformational changes along the polar filament structure, as suggested by Ngo et al. [159], is not considered. The boundaries between “a” and “b” blocks move stochastically without an energy cost, at a rate determined by the energy barrier between the two states and the temperature. (**D**) shows the binding of a specific ABP. Here, we consider a case in which ABP binding stabilizes the bound structure. The bound structure may involve a new actin conformation “c” along the conformation axis (left), which is a version of the “induced fit” model for binding of two proteins [167]. However, here we assume that the ABP binds one of the inherent conformations, say “a”, and stabilizes it (right). This is a version of the “conformational selection” model [167]. (**E**) When ABP molecules bind to an actin filament, as shown in (**C**), they would bind to the “a” protomers. Successive ABP binding would eventually convert the unstable “a” protomers at the boundaries (light green) to the stabilized “a” conformation (dark green). Then, the boundaries would move only into the “b” blocks, converting the unstable “b” conformation to the unstable “a” conformation, which would then be bound with the ABP and converted to the stable “a” conformation. The repetition of this process will expand the stable “a” block with the bound ABP, explaining the growth of the ABP cluster. Even if ABP binding induces a new conformation in the actin protomer, as illustrated on the left of (**D**), a similar scenario can be proposed. This scenario does not necessarily require the cooperative polymorphism of pure actin filaments shown in (**C**). Conversely, it becomes challenging to explain how ABP clusters grow without invoking the principle of cooperative polymorphism in the presence of ABPs, when the ABP molecules do not interact directly with each other along the actin filaments.

Single-molecule intramolecular Förster Resonance Energy Transfer (FRET) analysis also detected spontaneous polymorphism of actin filaments [136]. Notably, the FRET intensities consisted of two distinct populations, different from a broad single Gaussian distribution of helical twists [135,159]. The two FRET states spontaneously alternate with each other in the time scale of seconds, implying that they are metastable states in thermal equilibrium (Figure 5A). Interestingly, the abundance of one of the FRET states became higher than the other when actin filaments interacted with myosin V in the presence of ATP. This result suggests that interactions with certain ABPs can modulate the equilibrium, and we will come back to this point in the “Bound ABP-induced cooperative conformational changes” section. A more recent single-molecule intramolecular FRET analysis of actin employing different positions for fluorescent labeling suggested three to four distinct states [165]. Galkin et al. [166] further suggested that ADP-actin filaments contain six distinct conformations that cryo-EM can distinguish, though many subsequent cryo-EM studies failed to reproduce this intriguing result. The following sections will further elaborate on the concept that actin filaments have multiple metastable conformations in thermal equilibrium.

### 8.4. F-(4): Mechanical Strain

Actin filaments are flexible double-helical polymers, allowing them to be mechanically deformed in three distinct ways: stretching, bending, and twisting/untwisting. Naturally, the structure of individual actin protomers and their interactions with their neighbors should change in mechanically affected filament segments. The atomic details of those changes need to be better understood, but reports of the functional consequences are accumulating.

Micromanipulation experiments on isolated actin filaments demonstrated that actin filaments are relatively inextensible and the stiffness of a 1 µm filament with and without bound Tm was ~65 pN/nm and ~44 pN/nm, respectively [168], which is equivalent to ~0.25% of extension under isometric tension in muscle [169]. A coarse-grained molecular dynamics (MD) simulation suggested that 200 pN of stretching force induced two distinct changes: Filament lengths were stretched by 0.18% while the fluctuations were narrowed, and the twist angle along the short helix was reduced by 11% (untwisting), and the fluctuations were again narrowed [137]. Suppression of twisting fluctuation by tension was recently demonstrated experimentally [170].

Stretching actin filaments has distinct functional consequences. Pioneering work by Hayakawa et al. [54] discovered that stretched actin filaments are more resistant to the severing activity of cofilin in vitro (note, however, that a more recent study failed to detect such strain-sensitive actin binding of cofilin in a different experimental setup [171]). This can be explained by the result of the MD simulation mentioned above [137] if cofilin binds naturally supertwisted segments of actin filaments [163]. Hayakawa et al. also found that cofilin preferentially binds to relaxed stress fibers in cultured mammalian cells [54]. In those cells, actin filaments in stress fibers actively generating tension are stretched while preferential binding of cofilin to relaxed actin filaments would contribute to the removal of unused stress fibers in cells.

Measuring the binding affinity of myosin to actin filaments is challenging due to the inherent properties of myosin motors: they exhibit extremely weak actin affinity in the presence of physiological ATP concentrations and excessively strong affinity in its absence, making meaningful measurements difficult under either condition. To address this issue, two approaches can be employed. The first approach involves using very low ATP concentrations, as demonstrated by Tokuraku et al. [122] and discussed in detail in the “Bound ABP-Induced Cooperative Conformational Changes” section. The second approach is to utilize mutant myosin II with intermediate actin affinity in the presence of physiological ATP levels. Uyeda et al. adopted this strategy to detect mechanosensitivity of actin-myosin II binding under physiological conditions, employing two mutant S1 fragments of *Dictyostelium* myosin II that exhibited intermediate actin affinities under these conditions. By expressing GFP-fused versions of these mutant S1s in *Dictyostelium* cells, they showed that S1 preferentially binds to stretched actin filaments in vivo [3]. In cells, interaction of actin filaments with myosin II filaments generates tension in the posterior cortex of migrating cells or contractile rings in dividing cells so that this preference of myosin II S1 to bind to stretched actin filaments would form a positive feedback loop to stabilize the localized contractile activities (Figure 3B). The opposite preferences cofilin and myosin II show regarding the mechanical status of actin filaments are consistent with the idea that the opposite preferences contribute to complementary intracellular localizations of cofilin and myosin II in polarized *Dictyostelium* cells (Figure 3A) mentioned above.

In polarized *Dictyostelium* amoeba, myosin I is specifically localized along the leading edge [172]. This is distinct from myosin II, which is localized in the posterior cortex. Intriguingly, S1 of myosin I, with the same mutation as that used for myosin II, did not bind to stretched actin filaments in vivo [3]. In *Dictyostelium* amoeba, talin A is localized in the posterior cortex, and this is mediated by preferential binding of ABD of talin A to stretched actin filaments, whereas ABD of talin B, which is localized in the anterior region, does not bind to the stretched actin filaments [134]. Thus, the homologous ABDs of myosins and talins (S1 in the case of myosins) show different preferences for stretched and relaxed actin filaments.

α-Catenin mediates cell–cell binding in multicellular animals and shows complex actin-binding behaviors. Recent detailed structural analyses revealed that a short C-terminal extension of α-catenin ABD confers the mechanosensitivity of actin binding [173]. Tandem repeats of LIM domains, found in ABPs such as the FHL family of transcriptional factors, zyxin and paxillin in higher animals, also bind to tensed actin filaments in vivo and in vitro [174,175]. The binding of the FHL transcriptional factors to stretched actin filaments sequesters them in the cytoplasm and is believed to mediate mechanical strain-dependent regulation of transcription [174]. Zyxin and paxillin are also implicated in strain-dependent signal transduction. Intriguingly, paxillin-like protein in fission yeast also accumulates along stressed actin filaments, contractile rings in yeast and tensed stress fibers in mouse fibroblasts [175]. A recent MD simulation suggested that tension transiently breaks interprotomer contacts along one of the protofilaments, exposing cryptic binding sites for the LIM domain proteins [176]. Although this needs to be confirmed experimentally, an atomistic understanding of how a certain ABP recognizes tensed actin filaments appears close at hand.

Stretching forces to affect actin filaments can be generated either within the cell or from external sources. Internal sources of stretching forces acting on actin filaments can be found in acto-myosin contractile structures such as stress fibers [177], the posterior cortex of migrating cells [46] (Figure 3), and contractile rings of dividing animal and yeast cells [178]. External forces can affect intracellular actin filaments and evoke physiological responses. For example, stretching of the substratum applies tension to stress fibers and induces their reorientation [179]. When the tip of a micropipette pushed a peripheral region of a round and unpolarized keratocyte fragment, the pushed and dented portion became the posterior end of the fragment, and the fragment started to move away from the micropipette [180]. This may reflect tension-dependent recruitment of myosin II to the pushed area of the cortex.

Bending of actin filaments would cause opposite mechanical strains along the convex and concave sides of the curvature, as revealed by recent cryo-EM and modeling studies [151]. When polymerizing ends of filaments in the Arp2/3 complex-dependent branched meshwork of actin filaments in lamellipodia push the cell membrane outward, the reaction from the membrane pushes back the filaments. Because those filaments push the membrane diagonally due to the 70° branching angle, the reaction force bends the filaments, with the convex side facing outward. Risca et al. [181] demonstrated that Arp2/3 preferentially binds to the convex side of an actin filament, forming a new daughter filament on this side in vitro. This would bias the generation of new filaments on the outward-facing sides and contribute to suppressing the futile generation of new filaments growing away from the membrane in vivo. This is another case in which structural changes of actin filaments induced by mechanical strain could have physiological relevance.

Twisting and untwisting of the actin helix can be induced by molecular motors. When a formin dimer processively elongates an actin filament, it rotates around the actin long axis [182] and generates torque in the direction of untwisting the actin helix, which confers resistance to cofilin activities [183]. Vertebrate non-muscle myosin IIB (NMIIB) is a very slow motor and was shown to move processively for 4–5 steps of 5.4 nm along the helical protofilament [184]. This rotates the filament by ~90° around the long axis, twisting the helix on the plus-end side and untwisting the helix on the minus-end side of the bound motor. Those authors further found that fascin-mediated actin bundles disintegrate as they move over NMIIB-coated glass surfaces in vitro, presumably due to the azimuthal mechanical force produced by NMIIB.

### 8.5. F-(5): Bound ABP-Induced Cooperative Conformational Changes

Generally, when two protein molecules bind, each molecule is structurally impacted by its binding partner. It is thus natural that the binding of an ABP molecule to an actin protomer in a filament affects the structure of the bound actin protomer. As summarized in Table 1, ABP-induced conformational changes of actin protomers have been revealed by cryo-EM for α-catenin [120], cofilin [155] [11], ABD1 [185] and ABD2 [186] of fimbrin, scruin [187], and Lifeact (ABD of yeast ABP-140) [188]. ABP-induced conformational changes of actin filaments are also detected as alterations in helical pitch for cofilin [161], scruin [187], drebrin [125], and Rng2-CHD [189]. As described previously, single-molecule FRET analysis detected the structural impact of transient interaction with myosin V in the presence of ATP, observed as a shift in the equilibrium between the two metastable states [136].

The structural changes in the affected actin protomer would affect the structure of the four neighboring protomers that are in direct contact, and in turn, those structural changes may affect the structure of protomers further away. This could have profound consequences if segments of actin filaments can take several metastable conformations in equilibrium, as discussed before. In this situation, the structural impact of ABP binding to one actin protomer can affect multiple actin protomers in the filament, in a manner similar to a domino effect (Figure 5). This is a cooperative conformational change.

The first experimental suggestion that an ABP can cause conformational changes in actin filaments and that the conformational changes propagate to neighboring regions (cooperativity) was provided by a pioneering work by Oosawa et al. more than a half-century ago [190]. Those authors prepared fluorescent actin filaments and observed that fluorescence intensity was enhanced by the binding of muscle HMM in the absence of ATP (i.e., rigor binding). Moreover, fluorescence enhancement was saturated at a 1:5 molar binding ratio of HMM motor domain: actin protomer, strongly suggesting that the HMM-induced structural changes at the bound actin protomer are propagated cooperatively over ~four neighboring actin protomers. Various biophysical measurements also indicated cooperative conformational changes of actin filaments induced by muscle HMM or S1 in the absence of ATP [191,192,193,194,195]. Prochniewicz [195] found that S1 of muscle myosin II and S1-equivalent of myosin V cause different cooperative conformational changes to actin filaments. Intriguingly, cryo-EM studies on actin-myosin complexes failed to detect significant myosin-induced conformational changes in actin filaments [196,197]. It may be that the structural changes were simply too subtle to be detected by cryo-EM. Alternatively, the structural changes mainly altered the dynamic properties of the filaments, which cannot be detected by conventional cryo-EM analysis that determines the average structures. This latter scenario is consistent with the fact that most of the indirect biophysical analyses mentioned above measure dynamic properties of actin filaments [192,193,194,195].

More prominent ABP-induced structural changes of actin filaments are those induced by cofilin, as described above, and a number of cryo-EM studies have been conducted to understand the atomic detail [11,155,164]. The conformational change from the protomer’s structure in canonical actin filaments (F-form) to that in cofilin clusters (C-form) involves rigid body rotation between the outer and inner domains around two axes (Figure 1). The supertwisted conformational change is propagated to neighboring bare zones [155,198] on the minus-end side [138,159]. Tanaka et al. (2018) were able to propose a model for the propagation of the supertwisted conformational changes and why filament severing occurs preferentially at boundaries between the cluster and the bare zone [159,162,199,200].

Cofilin induces another, apparently different type of conformational changes to actin filaments that can be detected by time-resolved phosphorescence anisotropy [201] and differential scanning calorimetry [202,203]. It is notable that one molecule of bound cofilin reportedly changes the structure of ~100 actin subunits within a filament, but the structural details of these cooperative conformational changes have yet to be revealed. As with the case of HMM-induced cooperative conformational changes discussed above, these extremely long-range cofilin-induced changes detected by sensitive indirect biophysical measurements probably reflect changes in the filament dynamics or orientation of the amino acid side chains and are not amenable to current cryo-EM analysis.

Several other ABPs have also been shown to form clusters along actin filaments. For example, ABD of a neuronal ABP drebrin forms clusters along actin filaments, and this involves ~10% untwisting of the helical pitch [125,204]. ABD2 of fimbrin [124] and the motor domain fragments (S1 or HMM) of myosin II also form clusters. Using electron microscopy, Orlova and Egelman [121] observed that skeletal muscle HMM forms dense clusters along Ca^2+^-actin filaments. However, clusters did not form along physiological Mg^2+^-actin filaments, and the physiological relevance of this finding was unclear. Subsequently, as mentioned earlier, Tokuraku et al. [122] observed that GFP-HMM of *Dictyostelium* myosin II formed loose clusters along Mg^2+^-actin filaments only in the presence of low concentrations of ATP. It is unknown whether a low concentration of ATP is needed to realize moderate actin affinity or whether the ATP-bearing motor domain elicits some structural impact on the actin structure to generate a cluster.

For ABP cluster formation along actin filaments in the absence of external cues, a group of neighboring actin protomers must have an affinity for the ABP higher than the surrounding protomers. This can be achieved through the propagation of a conformational state along an actin filament (cooperative conformational changes) that is coupled with either of the two mechanisms for initial ABP binding: the “conformational selection” mechanism that assumes inherent conformational polymorphism in actin filaments, and the “induced fit” mechanism (Figure 5D). In the case of cofilin cluster formation, the initial actin binding presumably depends on the conformational selection mechanism to find a naturally supertwisted segment [138,163], and the subsequent, unidirectional cluster growth depends on cooperative conformational changes [159]. The cluster growth of fimbrin ABD-GFP [124] and HMM-GFP in the presence of 0.1 µM ATP [123] is also unidirectional, similar to cofilin clusters, and may involve a similar mechanism.

The conformational changes of actin protomers that recruit additional binding of the initial ABP may inhibit the binding of another ABP. In vitro cosedimentation experiments [53] demonstrated that cofilin binding to actin filaments partially inhibited the binding of myosin II S1 to bare sections of the actin filaments in the presence of ATP. Conversely, transient interaction of S1 to actin filaments in the presence of ATP strongly inhibited cofilin binding to the actin filaments. These mutual inhibitions cannot be due to direct competition for binding sites on actin molecules. Ngo et al. (2016) suggested that cooperative conformational changes induced either by cofilin or S1+ATP inhibited the binding of S1 or cofilin, respectively [53]. Regarding the cofilin-mediated inhibition of actin binding of myosin II, it may be possible to explain this by a shortened helical pitch near the cofilin clusters [159]. However, the mechanism of myosin-mediated inhibition of actin binding of cofilin is still elusive since structural information on actin filaments interacting with myosin II in the presence of ATP is quite limited, the exception being intramolecular FRET analyses [136,165] and maybe Toshio Yanagida’s early, intriguing observation that fluorescent actin filaments undergo vigorous bending motions in the presence of ATP and HMM [205]. Considering that cofilin is excluded from ATP- and ADP·Pi-actin filaments, it is possible that myosin motors in the presence of ATP may stimulate the exchange of actin-bound ADP with ATP in solution. Moos and Eisenberg [206] showed that muscle myosin II filaments stimulated ADP release from actin filaments. Still, this classic study found that HMM did not have such activity, so further studies are warranted.

Actin binding of cofilin displaces Tm [207,208], and since binding sites of cofilin and Tm do not overlap on actin protomers, this inhibition likely involves cofilin-induced conformational changes of actin filaments [161]. Conversely, Tm binding stiffens actin filaments, as demonstrated by light scattering [209] and persistence length measurements [160]. Such restrictions on the bending motions and presumably twisting/untwisting motions of the actin filaments might impact the binding of other ABPs with a preference for a particular curvature or helical pitch.

Mutually cooperative and exclusive interactions among groups of ABPs are also known in the fission yeast system. Among the three actin structures in yeast cells, actin patches are assembled by Arp2/3 complexes and contain fimbrin and cofilin Adf1. However, another actin crosslinker α-actinin, as well as Tm, are excluded from actin patches. Contractile rings, assembled by a formin Cdc12, contain α-actinin and Tm but lack fimbrin. The exclusion of Tm from actin patches depends on fimbrin. This function of fimbrin seems to rely on cooperative conformational changes in actin filaments induced by fimbrin rather than its crosslinking activity. This is supported by the observation that fimbrin’s ABD2, which cannot bundle actin filaments, is sufficient to localize to actin patches and displace Tm [210]. Separate in vitro studies have further demonstrated that fimbrin ABD2 induces conformational changes in actin filaments [186] in a cooperative manner [124]. Christensen et al. successfully replicated these cooperative and mutually exclusive actin-binding behaviors among α-actinin, Tm, fimbrin, and cofilin in vitro. Interestingly, despite both the ABD of α-actinin and the ABD2 of fimbrin being composed of tandem CHDs, α-actinin and Tm collaborate to bind actin filaments and displace fimbrin [115]. This suggests that the ABD of α-actinin and/or Tm induces cooperative conformational changes in actin filaments, stabilizing their own binding while destabilizing fimbrin binding.

Nucleation factors at filament ends are also known to induce long-ranged cooperative conformational changes to the actin filaments that they assembled. For example, a formin dimer bound at the plus end of an actin filament affects the conformation of the entire actin filament in such a way that the changes can be detected by fluorescence-lifetime and anisotropy decay experiments [211], which may accompany increased flexibility of the filament [212]. As already discussed in “Tm isoform-dependent selection of ABPs”, Johnson et al. [105] demonstrated that two formin isoforms in the fission yeast specify which Tm isoform to bind to on the actin filaments they generate.

In terms of the functional influence the Arp2/3 complex exerts on the filament that it polymerizes, a reconstitution study by Michelot et al. [213] is worth mentioning. Actin patches, actin cables and contractile rings are the three actin cytoskeletal systems in budding yeast, and Arp2/3 is responsible for assembling actin patches (two formins assemble the other two). Intriguingly, Michelot et al. (2010) [213] observed that only a subset of yeast ABPs associated with actin filaments polymerized by the Arp2/3 complex in vitro, while proteins typically recruited to actin cables and contractile rings were excluded. Building on this work, Homa et al. found that fimbrin and Tm specifically bind to Arp2/3- and formin-dependent actin filaments, respectively, when all four components are mixed in a single solution [214]. These findings provide insight into how specific ABPs accumulate in actin patches. Although the involvement of Arp2/3- and formin-induced conformational changes in actin filaments has been speculated [214], the precise mechanism underlying this specificity remains unclear. Based on their earlier results, Michelot et al. then proposed that “actin filaments acquire an identity at birth” [4].

A critical piece of missing information regarding the hypothesis that ABP-induced conformational changes of actin filaments drive functional differentiation of actin filaments is the demonstration that actin filaments in cells are polymorphic.

Finally, negative and positive impacts of actin conformational changes on the motility of myosin II in vitro are discussed. During in vitro motility assays, the sliding speeds of actin filaments are primarily determined by the type of myosin, and not by actin (e.g., [215], but see [150,216,217] for examples of actin-dependent modulation of actin-myosin motilities). Intriguingly, very sparse binding of a motor activity-less G680V mutant myosin S1 [218] to actin filaments doubled the speed of actin filaments driven by skeletal muscle HMM absorbed on the surface [219]. In contrast, sparse binding of the ABD of a yeast IQGAP actin-binding protein Rng2 (Rng2CHD) to actin filaments potently inhibited the motility by muscle HMM [189]. Although the structural impact actin filaments receive from the binding of G680VS1 in the presence of ATP is unknown, sparse binding of Rng2CHD was shown to cooperatively shorten the helical pitch of actin filaments [189]. Obviously, certain cooperative conformational changes in actin filaments significantly affect the actin-myosin interaction positively and negatively in an unknown way. This idea is supported by motility inhibition by three mutations that likely affect conformational changes of actin (M47A in D-loop [220] and G146V [221] and K336I [222] in the hinge between subdomains 1 and 3). Chemical intramolecular crosslinking of actin using two different crosslinkers [223,224], which would also impede conformational changes of actin protomers, also strongly inhibited actin sliding on muscle HMM-coated surfaces. Elucidating how myosin affects the conformation of actin filaments and how the conformational changes in actin filaments affect the motor activity of myosin would shed new light on the unexplored aspect of force generation mechanism by myosin.

**Table 1:** Conformational changes of actin filaments induced by ABP binding.

ABP	Detected by				
	Cryo-EM	AFM	Cooperative Binding (Cluster Formation)	Cooperative Binding (Binding Ratio)	Other Biophysical Methods
α-catenin	[120]				
Cofilin (short range)	[11,155,161]	[159]	[159,161,162]	[161,225,226,227]	[228,229]
Cofilin (long range)					[201,202,203]
Coronin 1-C				[118]	
Drebrin		[125,204]			
ABD of dystrophin				[119]	
ABD1 of fimbrin	[185]				
ABD2 of fimbrin	[186]		[124]		
Formin					[211,212]
Gelsolin					[230]
Lifeact	[188]				
Myosin II motor (−ATP)			[121]		[190,191,192,193,194]
Myosin II motor (+ATP)			[122,123]		
Myosin V +ATP					[136]
ABD of Rng2		[189]			
Tm				[84]	
Jasplakinolide *	[14]				
Phalloidin *	[13,14]				

* Jasplakinolide and phalloidin are not proteins but are listed here as references.

## 9. Fundamental vs. Lineage-Specific Regulatory Mechanisms and the Hierarchy Among Them

Several molecular mechanisms regulate which ABPs interact with a specific set of actin filaments, leading to the functional differentiation of actin filaments in a cell. Those mechanisms are not mutually exclusive but are unequal in their significance in several ways. For instance, some mechanisms can directly or indirectly determine ABP binding, while others only modulate or enhance the process, determined by the upstream factor. From the perspective of this review, some are conserved among many eukaryotic lineages, implying that they are derived from LECA and are thus fundamental, while others are restricted to a specific lineage of complex multicellular organisms. Table 2 summarizes the regulatory mechanisms’ hierarchy and whether they are fundamental or lineage-specific.

External chemical signals and mechanical stimuli trigger localized actin-dependent processes and cell polarization. Cells often spontaneously develop polarity in the absence of external cues. The most notable examples are bi-polarization formed by signals from mitotic spindles and polarity generation in fertilized eggs. However, spontaneous cell polarization can occur without such obvious distinct intracellular cues. For this, thermal and stochastic fluctuation of the activity of ABP, its upstream regulator, or the structure of actin filaments to produce a patch of a particular conformation may play a role. Once an actin nucleator is committed to assembling an actin filament, or an existing actin filament is destined to change its function by changing the binding ABP partner, the next level of regulatory mechanisms participate by recruiting ABP that actually modify the actin functions such as motor proteins, crosslinkers which include those that link actin filaments and those that link actin filaments to other structures and membranes, stabilizers, and severing factors and cappers. We propose that a primary mechanism for this level of regulation is the set of cooperative conformational changes induced by ABPs, which is fundamental. Aging involving ATP hydrolysis and Pi release is another fundamental mechanism that plays a critical, autonomous regulatory role at this level. Presumably, the ancestral eukaryotic actin in LECA already harnessed those regulatory processes, which made most of the subsequent changes in the actin primary structure intolerable [232].

Tm-mediated, actin-isoform-specific, and PTM-dependent regulations also play important roles in certain lineages, although these are not fundamental. Those multiple intricate regulatory mechanisms collaborate with others to ensure efficient and robust execution of a number of actin-dependent cellular functions. A mutually exclusive relationship between myosin II and ADF/cofilin is worth mentioning here. As described in the “Tropomyosin (Tm) isoform-dependent selection of ABPs” section, overexpression of the Tm3.1 isoform recruited myosin II to stress fibers while inhibiting the binding of ADF/cofilin to the actin filaments. In contrast, overexpression of the Tm1.12 isoform promoted ADF/cofilin binding to actin filaments that do not contain myosin II [87,88]. However, in vitro binding experiments using purified actin, myosin II fragments, and cofilin but without Tm demonstrated that cofilin binding to actin filaments inhibits myosin II binding and myosin II binding inhibits cofilin binding in the presence of ATP, in a manner dependent on cooperative conformational changes of actin filaments [53]. Thus, it appears that, in this case, the differential regulation by Tm3.1/Tm1.12 reinforces the fundamental, intrinsic regulatory functions of actin filaments, rather than creating a novel form of regulation. Proper localizations of myosin II and V in mutant yeasts with mislocalized formins and Tm isoforms [105] may also suggest ancillary roles of Tm isoforms.

## 10. Conclusions

The eukaryotic actin filament system is regulated by multiple mechanisms. Some appear to be inherited from LECA, and we called them “fundamental”. Other, lineage-specific regulatory mechanisms evolved later, particularly in complex multicellular organisms, presumably in such a manner that they would enhance or complement the functions of the fundamental mechanisms. There are hierarchical regulatory relationships between certain pairs of these mechanisms and more studies are needed to understand the relationship between the regulatory mechanisms. Needless to say, much more research efforts are also needed to unveil the molecular mechanism of each regulatory mechanism. Researchers have been studying actin functions with remarkable ingenuity, employing a wide range of approaches and leveraging recent advances in microscopic techniques such as cryo-EM and total internal reflection fluorescence (TIRF) microscopy. However, for a truly atomistic understanding of regulatory mechanisms, MD simulations likely need to be incorporated more extensively, while HS-AFM could serve as a crucial bridge between cryo-EM and light microscopy studies.

## Figures and Tables

**Table 2:** Speculative classification of actin-regulatory mechanisms.

Mechanism	Hierarchy	Fundamental vs. Lineage-Specific *
Local biochemical regulation of actin nucleators	High	Fundamental **
Local biochemical regulation of side-binding and capping ABPs	High	Mixed?
Differences in dissociation rates	Low	Fundamental?
Actin isoform-dependent selection of binding ABPs	Low	Lineage-specific
Tm isoform-dependent selection of binding ABPs	Low	Lineage-specific
Physical geometry-dependent selection of ABPs	Low	Lineage-specific
Post-translational modification of actin	Low	Lineage-specific
Aging	High	Fundamental
Thermal and stochastic fluctuations	High ***	Fundamental
Mechanical strain	High	Fundamental
Bound ABP-induced cooperative conformational changes of actin filaments	Low	Fundamental ****

The hierarchy between two regulatory mechanisms can be determined or inferred from experimental evidence. High-hierarchy mechanisms are the upstream regulators that employ low-hierarchy mechanisms to ultimately determine the function of actin filaments. Fundamental mechanisms are those employed by LECA and can be inferred from the distribution in the extant eukaryotic lineages with reasonable confidence. In assessing the fundamental nature of each actin regulatory mechanism, we did not consider the potential contribution of lateral or horizontal gene transfer. While lateral gene transfer among eukaryotes is less common than among prokaryotes [231], it is challenging to entirely dismiss the possibility that such transfer played a role in spreading specific actin regulatory mechanisms within Eukarya—particularly among the close descendants of LECA, about which we know very little. However, we can confidently exclude the contribution of lateral gene transfer in the dissemination of regulatory mechanisms involving multiple components. Examples include formins and their upstream regulators, as well as the Arp2/3 complex, which comprises seven subunits along with NPFs. It is highly improbable that the entire set of genes encoding these multiple components was transferred laterally between eukaryotes. *: This judgment depends primarily on the properties of muscle α actin and the distribution of various ABPs in Eukarya, and needs to be validated by future comparative studies using actins from various extant species. **: Formins and the Arp2/3 complex are present in the majority of eukaryotes, whereas Spire, Cordon-bleu, leiomodin, and Chup1 are lineage-specific. As a result, regulatory mechanisms involving these lineage-specific nucleators are not considered fundamental. ***: Thermal and stochastic fluctuations can directly affect ABP binding, as in the case of cofilin binding [138], but can also activate other high-hierarchy mechanisms. ****: We propose that actin filaments in LECA were regulated by cooperative conformational changes induced by ABP that LECA possessed. However, a number of lineage-specific regulations mediated by ABP-induced cooperative conformational changes presumably emerged during subsequent evolution.
